# Supplementary material for: Diversity and antibiotic susceptibility profiles of bacterial isolates from wound infections in patients at the surgical unit of Kisii teaching and referral hospital, Kenya
Source: PLoS One. 2025 Jun 17;20(6):e0326048. doi: 10.1371/journal.pone.0326048 (PMC12173245; doi:10.1371/journal.pone.0326048)
Supplement: Attachment — Submitted filename: PONE-D-25-04326 reviewed AAO.pdf [file pone.0326048.s002.pdf]

PLOS ONE

Diversity And Antibiotic Susceptibility Profiles of Bacterial Isolates from Wound Infections in Patients at the Surgical Unit of Kisii Teaching and Referral Hospital, Kenya

--Manuscript Draft--

**Manuscript Number:**	PONE-D-25-04326
**Article Type:**	Research Article
**Full Title:**	Diversity And Antibiotic Susceptibility Profiles of Bacterial Isolates from Wound Infections in Patients at the Surgical Unit of Kisii Teaching and Referral Hospital, Kenya
**Short Title:**	Diversity And Antibiotic Susceptibility Profiles of Bacterial Isolates from Wound Infections
**Corresponding Author:**	Samson Onsando, C.O<br>Jomo Kenyatta University of Agriculture and Technology<br>Nairobi, KENYA
**Keywords:**	Antimicrobial resistance; Wound infections; Bacterial isolates; Antibiotic susceptibility; Multidrug resistance; Empirical antibiotic therapy.
**Abstract:**	Antimicrobial resistance (AMR) poses a growing threat to global health, with wound infections serving as significant reservoirs for multidrug-resistant bacteria. Understanding the socio-demographic, clinical, and microbiological characteristics of wound infections is critical for effective intervention strategies in resource-limited settings. Thus, this study aimed to evaluate the diversity, bacterial profiles, and resistance patterns of wound infections at Kisii Teaching and Referral Hospital. To do this, a total of 118 bacterial isolates from wound swabs were collected from 149 patients who met the eligibility criteria, from 15th June 2024 to 30th October 2024. These samples were cultured and analysed using standard microbiological techniques and antibiotic susceptibility testing according to the Clinical and Laboratory Standards Institute guidelines. Resistance genes associated with Vancomycin resistance in Staphylococcus aureus and Staphylococcus xylosus were also investigated using polymerase chain reaction (PCR) with appropriate primers and agarose gel electrophoresis. The results revealed a predominance of male patients (53.02%) and wounds predominantly on lower limbs (42.29%). Escherichia coli (27.97%), Enterobacter gergoviae (16.95%), Staphylococcus aureus (15.25%), and Staphylococcus xylosus (15.25%) were the most frequently isolated pathogens. Alarmingly high resistance rates were observed against ACCESS antibiotics, including Cotrimoxazole-Trimethoprim (97%), Ampicillin (92%), and Streptomycin (85%). Resistance to Watch and Reserve antibiotics, including Vancomycin (29%) and Ceftriaxone (33%), was also documented, particularly among Gram-positive isolates such as Staphylococcus aureus and Gram-negative pathogens like Pseudomonas aeruginosa. Resistance genes (VanA, VanB, VanC) were detected in S. aureus and S. xylosus, underscoring the role of genetic mechanisms in resistance propagation. These findings highlight the critical need for targeted antimicrobial stewardship programmes and strengthened infection prevention measures in high-burden hospital settings, such as Kisii Teaching and Referral Hospital. This study underscores the importance of local resistance data in guiding empirical therapy and informs policies to mitigate AMR in resource-limited contexts. Moreover, enhanced surveillance and research into alternative therapies are crucial to safeguarding the efficacy of life-saving antibiotics and improving patient care outcomes.
**Order of Authors:**	Samson Onsando
	Andrew Nyerere, PhD
	Moses Njire, PhD
	Wycliffe Masanta, PhD
	Gervason Moriasi, Ph.D
**Additional Information:**	

Powered by Editorial Manager® and ProduXion Manager® from Aries Systems Corporation

Question	Response
**Financial Disclosure**<br><br>Enter a financial disclosure statement that describes the sources of funding for the work included in this submission. Review the submission guidelines for detailed requirements. View published research articles from *PLOS ONE* for specific examples.<br><br>This statement is required for submission and **will appear in the published article** if the submission is accepted. Please make sure it is accurate.<br><br>**Funded studies**<br>Enter a statement with the following details:<br>• Initials of the authors who received each award<br>• Grant numbers awarded to each author<br>• The full name of each funder<br>• URL of each funder website<br>• Did the sponsors or funders play any role in the study design, data collection and analysis, decision to publish, or preparation of the manuscript?<br><br>Did you receive funding for this work?	The author(s) received no specific funding for this work.
**Competing Interests**<br><br>Use the instructions below to enter a competing interest statement for this submission. On behalf of all authors, disclose any competing interests that could be perceived to bias this work—acknowledging all financial support and any other relevant financial or non-financial competing interests.<br><br>This statement is required for submission and **will appear in the published article** if the submission is accepted. Please make sure it is accurate and that any funding sources listed in your Funding Information later in the submission form are also	The authors have declared that no competing interests exist

declared in your Financial Disclosure statement.<br><br>View published research articles from *PLOS ONE* for specific examples.<br><br>**NO authors have competing interests**<br><br>Enter: *The authors have declared that no competing interests exist.*<br><br>**Authors with competing interests**<br><br>Enter competing interest details beginning with this statement:<br><br>*I have read the journal's policy and the authors of this manuscript have the following competing interests: [insert competing interests here]*<br><br>* typeset	
**Ethics Statement**<br><br>Enter an ethics statement for this submission. This statement is required if the study involved:<br><br>• Human participants<br>• Human specimens or tissue<br>• Vertebrate animals or cephalopods<br>• Vertebrate embryos or tissues<br>• Field research<br><br>Write "N/A" if the submission does not require an ethics statement.<br><br>General guidance is provided below. Consult the submission guidelines for detailed instructions. **Make sure that all information entered here is included in the Methods section of the manuscript.**	Ethical approval was obtained from the KTRH Ethics and Research Committee, University of Eastern Africa Baraton (REC: UEAB/ISERC/08/05/2024). Written informed consent was obtained from all participants or their legal guardians. Confidentiality of patient information was maintained throughout the study, and all procedures adhered to the Declaration of Helsinki principles.

Powered by Editorial Manager® and ProduXion Manager® from Aries Systems Corporation

**Format for specific study types**<br><br>**Human Subject Research (involving human participants and/or tissue)**<br>• Give the name of the institutional review board or ethics committee that approved the study<br>• Include the approval number and/or a statement indicating approval of this research<br>• Indicate the form of consent obtained (written/oral) or the reason that consent was not obtained (e.g. the data were analyzed anonymously)<br><br>**Animal Research (involving vertebrate animals, embryos or tissues)**<br>• Provide the name of the Institutional Animal Care and Use Committee (IACUC) or other relevant ethics board that reviewed the study protocol, and indicate whether they approved this research or granted a formal waiver of ethical approval<br>• Include an approval number if one was obtained<br>• If the study involved *non-human primates*, add *additional details* about animal welfare and steps taken to ameliorate suffering<br>• If anesthesia, euthanasia, or any kind of animal sacrifice is part of the study, include briefly which substances and/or methods were applied<br><br>**Field Research**<br><br>Include the following details if this study involves the collection of plant, animal, or other materials from a natural setting:<br>• Field permit number<br>• Name of the institution or relevant body that granted permission	
**Data Availability**<br><br>Authors are required to make all data underlying the findings described fully available, without restriction, and from the time of publication. PLOS allows rare exceptions to address legal and ethical concerns. See the PLOS Data Policy and FAQ for detailed information.	Yes - all data are fully available without restriction

A Data Availability Statement describing where the data can be found is required at submission. Your answers to this question constitute the Data Availability Statement and **will be published in the article**, if accepted.<br><br>**Important:** Stating 'data available on request from the author' is not sufficient. If your data are only available upon request, select 'No' for the first question and explain your exceptional situation in the text box.<br><br>Do the authors confirm that all data underlying the findings described in their manuscript are fully available without restriction?	
**Describe where the data may be found in full sentences. If you are copying our sample text, replace any instances of XXX with the appropriate details.**<br><br>• If the data are **held or will be held in a public repository**, include URLs, accession numbers or DOIs. If this information will only be available after acceptance, indicate this by ticking the box below. For example: *All XXX files are available from the XXX database (accession number(s) XXX, XXX.).*<br>• If the data are all contained **within the manuscript and/or Supporting Information files**, enter the following: *All relevant data are within the manuscript and its Supporting Information files.*<br>• If neither of these applies but you are able to provide **details of access elsewhere**, with or without limitations, please do so. For example:<br><br>*Data cannot be shared publicly because of [XXX]. Data are available from the XXX Institutional Data Access / Ethics Committee (contact via XXX) for researchers who meet the criteria for access to confidential data.*<br><br>*The data underlying the results presented in the study are available from (include the name of the third party*	All relevant data are within the manuscript and its Supporting Information files.

and contact information or URL).<br>• This text is appropriate if the data are owned by a third party and authors do not have permission to share the data.<br><br>* typeset	
Additional data availability information:	

Manuscript

Click here to access/download;Manuscript;Manuscript-M Onsando et al Jan2025-PLoS (2).docx

Diversity And Antibiotic Susceptibility Profiles of Bacterial Isolates from Wound Infections in Patients at the Surgical Unit of Kisii Teaching and Referral Hospital, Kenya

Samson Onsando[1,2] ORCID, Andrew Nyerere[2] ORCID, Moses Njire[3] ORCID, Wycliffe Masanta [4] ORCID, and Gervason Moriasi [5,6] ORCID.

[1]Kisii Teaching and Referral Hospital, P.O. Box 92-40200 Kisii, Kenya.

[2]Department of Medical Microbiology, College of Health Sciences, Jomo Kenyatta University of Agriculture and Technology, P.O. Box 62000 – 00200 Nairobi, Kenya.

[3]Department of Botany, Jomo Kenyatta University of Agriculture and Technology, P.O. Box 62000 – 00200 Nairobi, Kenya.

[4]Department of Medical Microbiology, School of Medicine, Maseno University, Private Bag, Maseno, Kenya

[5]Department of Medical Biochemistry, Medical School, Mount Kenya University, PO BOX 342-0100, Thika, Kenya

[6]Department of Biochemistry, Microbiology, and Biotechnology, School of Pure and Applied Sciences, Kenyatta University, PO BOX 43844-00100, GPO, Nairobi, Kenya.

Correspondence: Samson Onsando; Email: samonsando@gmail.com

Abstract

Antimicrobial resistance (AMR) poses a growing threat to global health, with wound infections serving as significant reservoirs for multidrug-resistant bacteria. Understanding the socio-demographic, clinical, and microbiological characteristics of wound infections is critical for effective intervention strategies in resource-limited settings. Thus, this study aimed to evaluate the diversity, bacterial profiles, and resistance patterns of wound infections at Kisii Teaching and Referral Hospital. To do this, a total of 118 bacterial isolates from wound swabs were collected from 149 patients who met the eligibility criteria, from 15th June 2024 to 30th October 2024. These samples were cultured and analysed using standard microbiological techniques and antibiotic susceptibility testing according to the Clinical and Laboratory Standards Institute guidelines. Resistance genes associated with Vancomycin resistance in *Staphylococcus aureus* and *Staphylococcus xylosus* were also investigated using polymerase chain reaction (PCR) with appropriate primers and agarose gel electrophoresis. The results revealed a predomin[illegible]e of male patients (53.02%) and wounds predominantly on lower limbs (42.29%). *Escheric[illegible] coli* (27.97%), *Enterobacter gergoviae* (16.95%), *Staphylococcus aureus* (15.25%), and *Staphylococcus xylosus* (15.25%) were the most frequently isolated pathogens. Alarmingly high resistance rates were observed against ACCESS antibiotics, including Cotrimoxazole-Trimethoprim (97%), Ampicillin (92%), and Streptomycin (85%). Resistance to Watch and Reserve antibiotics, including Vancomycin (29%) and Ceftriaxone (33%), was also documented, particularly among Gram-positive isolates such as *Staphylococcus aureus* and Gram-negative pathogens like *Pseudomonas aeruginosa*. Resistance genes (*Van*A, *Van*B, *Van*C) were detected in *S. aureus* and *S. xylosus*, underscoring the role of genetic mechanisms in resistance propagation. These findings highlight the critical need for targeted antimicrobial stewardship programmes and strengthened infection prevention measures in high-burden hospital settings, such as Kisii Teaching and Referral Hospital. This study underscores the importance of local resistance data in guiding empirical therapy and informs policies to mitigate AMR in resource-limited contexts. Moreover, enhanced surveillance and research into alternative therapies are crucial to safeguarding the efficacy of life-saving antibiotics and improving patient care outcomes.

Keywords: Antimicrobial resistance; Wound infections; Bacterial isolates; Antibiotic susceptibility; Multidrug resistance; Empirical antibiotic therapy.

Introduction

Wound infections pose a significant global health challenge, particularly in resource-limited settings where healthcare infrastructure may be inadequate [1]. Recent estimates suggest that approximately 7% of hospitalised patients in low- and middle-income countries develop wound infections, with even higher rates in surgical wards [2,3]. The diversity of bacterial pathogens associated with wound infections is extensive and dynamic, influenced by factors such as the nature of the wound, the patient's immune status, and the surrounding environment[4,5]. These infections can delay wound healing, increase morbidity, and, in severe cases, lead to systemic conditions such as sepsis [3]. Effective management of wound infections requires a thorough understanding of the bacterial flora involved and their antibiotic sensitivity profiles [5,6]. Without such data, clinicians face challenges in selecting effective empirical therapies [7].

The surgical unit, as a critical healthcare domain, frequently manages wound infections stemming from trauma, surgical procedures, burns, and chronic conditions such as diabetic foot ulcers [8,9]. Globally, trauma-related wound infections account for nearly 30% of admissions in surgical units and are often associated with higher incidences of morbidity and debility [10]. Infections in such settings are often polymicrobial, involving Gram-positive, Gram-negative, and occasionally anaerobic bacteria, including *Staphylococcus aureus*, *Pseudomonas aeruginosa, Escherichia coli, Klebsiella* spp., and *Proteus spp*. among others [11,12]. However, the prevalence and diversity of these pathogens can vary geographically and temporally, necessitating localised studies to inform evidence-based interventions [13,14]. Furthermore, environmental factors and hospital infection control practices significantly influence the microbial landscape, making continuous surveillance critical [15].

The rising prevalence of antimicrobial resistance (AMR) further complicates the management of wound infections [16]. According to the World Health Organisation, over 4.71 million deaths annually are attributed to bacterial infections, with about 1.14 million deaths attributed to bacterial AMR, with a substantial proportion linked to bacterial infections [17]. Misuse and overuse of antibiotics in clinical settings have accelerated the emergence of resistant strains, including methicillin-resistant *Staphylococcus aureus* (MRSA), extended-spectrum beta-lactamase (ESBL)-producing Enterobacteriaceae, and carbapenem-resistant *Pseudomonas* and *Acinetobacter* species [18]. This resistance compromises therapeutic outcomes, leading to

prolonged hospital stays, increased healthcare costs, and higher mortality rates [16,18]. Alarmingly, studies report resistance rates exceeding 60% for some commonly used antibiotics in sub-Saharan Africa, underscoring the urgency of evidence-based and targeted interventions [17,19]. Previous studies in Kenya have highlighted the presence of multidrug-resistant bacteria in various clinical samples[20–22], but data specific to wound infections in this region remain scarce, hindering the development of tailored antimicrobial stewardship strategies and effective infection control measures.

Therefore, this study aimed at characterising the diversity of bacterial isolates from wound infections in patients attending the surgical unit at Kisii Teaching and Referral Hospital (KTRH). Additionally, it evaluated the antibiotic sensitivity profiles of these isolates to guide empirical treatment strategies and inform antimicrobial stewardship programmes. This study's findings will contribute to improved patient outcomes and reduce the burden of wound infections in within the region, and beyond. By providing a robust evidence base, this research can also support policy development and resource allocation for managing AMR in similar healthcare settings.

Methodology

Study site

This study was conducted at Kisii Teaching and Referral Hospital (KTRH), which is located in Southwestern Kenya and serves as a major referral centre in Nyanza province region, over a six-month period, from May to November 2024. KTRH has a bed capacity of 700 with an occupancy rate of up to 150 %. It serves over 6 million people mainly from South Nyanza, and parts of the Rift Valley, and the northern parts of Tanzania, providing clinical, oncology, laboratory, critical care, radiology, rehabilitation and mental, gynaecological and reproductive health, paediatric, surgical, among other specialised care services. The hospital's surgical unit manages a high volume of patients with diverse wound types, providing an ideal setting to investigate the bacterial spectrum and resistance patterns. The surrounding rural and peri-urban areas contribute a diverse patient demographic, reflecting varying socioeconomic and environmental factors associated with injuries and AMR.

Study Design

This study adopted a descriptive cross-sectional design to characterise the diversity of bacterial isolates and determine their antibiotic sensitivity profiles.

Study Population and Sample Size

The study targeted patients attending the surgical unit at KTRH with clinical signs of wound infections, including purulent discharge, erythema, swelling, or delayed wound healing. A total of 149 patients meeting the predefined inclusion criteria were enrolled in the study from 15th June 2024 to 30th October 2024. The formula described previously [23] (Eq.1), as adapted by Maveke et al. [22] was used to determine the ideal sample size for this study.

$$n = \frac{\left(Z\alpha\sqrt{(2pq)} - Z\beta\sqrt{p1q1 + p2q2}\right)^2}{(p1 - p2)^2} \ldots\ldots\ldots\ldots\ldots\ldots\ldots..(Eq.1)$$

Where n = sample size; Zα = the statistic confidence level (1.96); Zβ = type 2 error at 20% (–0.84); p = 0.47, which was the estimated pooled proportion of ESBL producing *Enterobacteriaceae* established in East African hospitals [24]; p1= proportion at risk in the case group which was assumed to be 60% (0.6) for this study; p2= proportion at risk in the control group which was assumed to be 40% (0.4); p1-p2 = effect size which was assumed to be 20% (0.2).

Therefore,

$$n = \frac{[1.96\sqrt{(2 \times 0.47 \times 0.53)} - -0.84\sqrt{(0.6 \times 0.4) + (0.4 \times 0.6)}]^2}{(0.6 - 0.4)^2}$$

$$= 96.57 \text{ » } 100 \text{ samples}$$

To account for potential non-responses or incomplete data, the sample size was adjusted to 149 participants based on the finite population correction.

Inclusion and exclusion criteria

Patients were eligible for inclusion if they presented with clinically infected wounds and had not received antibiotics within the previous 48 hours. Exclusion criteria included patients with systemic infections unrelated to wounds, those on prolonged immunosuppressive therapy, and individuals unwilling to provide informed consent.

Wound site preparation and sample collection

Wounds were initially rinsed with sterile normal saline to minimise contamination as recommended previously [25]. For wounds covered with dressings, the dressing was carefully removed, and the area was thoroughly cleansed with sterile normal saline to eliminate surface exudates, necrotic tissue, and skin flora. The samples, including pus, purulent exudates, and other secretions, were collected aseptically using sterile cotton swabs (Deltalab)[26]. Adequate pressure was applied during swabbing to ensure sufficient specimen retrieval from the wound

bed, without causing further injury or pain to the patient. All specimens were transported to the KTRH Microbiology Laboratory within 30 minutes, logged by laboratory personnel involved in the study and immediately processed for microbial culture and identification.

Microbiological culturing and identification procedure

The collected samples were cultured on Sheep Blood Agar (BA), MacConkey Agar (MCA), Ashdown Agar (ADA), and Cystine Lactose Electrolyte Deficient (CLED) media, prepared according to standard protocols [27–29]. Briefly, commercially available powdered media were reconstituted with triple-distilled water and sterilised by autoclaving at 121°C for 15 minutes, following the manufacturer's guidelines. Inoculation onto culture media was performed using the streak plate method, where a sterile wire loop was aseptically used to streak the specimens, to ensure the formation of discrete colonies. The inoculated plates were incubated under both aerobic and anaerobic conditions at 37°C for 24 hours, with extended incubation of up to five days for Ashdown Agar to allow the growth of slow-growing organisms. Subculturing under the same conditions was undertaken to obtain pure colonies. The most predominant colonies were isolated using sterile swabs for further characterisation. Besides, colony morphology was recorded, and a series of biochemical and phenotypic including Gram staining, oxidase, catalase, and coagulase tests, as well as the API test systems (API 20 E, API 20 NE, and API STAPH) were performed for precise microbial identification. Further confirmation of bacterial species was performed using an automated VITEK® 2 compact system[30]. The isolates were preserved at -20°C for downstream applications, including antimicrobial susceptibility testing, DNA extraction, and polymerase chain reaction (PCR) analysis.

Antibiotic susceptibility testing

The antibiotic susceptibility of bacterial isolates was evaluated using the Kirby-Bauer disk diffusion assay, according to Clinical and Laboratory Standards Institute (CLSI) guidelines [31]. The bacterial isolates were inoculated onto Mueller-Hinton agar plates using a standard procedure to achieve uniform distribution. Antibiotic disks (Octo Discs) including Ampicillin (25µg), Tetracycline (100µg), Co-trimoxazole (Sulphathiazole/Trimethoprim) (23.75/1.25µg), Nitrofurantoin (200µg), Nalidixic acid (30µg), Streptomycin (25µg), and Gentamicin (10 µg), Vancomycin(30µg), Ceftriaxone(30µg), Linezolid(30µg), and Ofloxacin (5µg), were carefully placed on the agar surface, and plates were incubated under optimal conditions to promote bacterial growth and inhibition. Following incubation, the diameters of the inhibition zones

were measured precisely using a calibrated ruler, recorded, interpreted according to CLSI breakpoints, and categorised as susceptible, intermediate, or resistant [27,31,32].

DNA extraction, quantification, and analysis

DNA extraction

Bacterial DNA was extracted using the Quick-DNA™ Miniprep Kit (Zymo Research) according to the manufacturer's protocol [33]. Briefly, 500 µL of Genomic Lysis Buffer was added to the cell pellet, followed by vortexing for 5 seconds to lyse the cells. The mixture was incubated at room temperature for 10 minutes before being transferred to a Zymo-Spin™ IICR column placed in a collection tube. The assembly was centrifuged at 10,000 rpm for 1 minute, and the flow-through was discarded. After that, the Zymo-Spin™ IICR column was transferred to a fresh collection tube, and 200 µL of DNA Pre-Wash Buffer was added. The tube was centrifuged at 10,000 rpm for 1 minute, and the flow-through was discarded. Subsequently, 500 µL of DNA Wash Buffer was added to the column and centrifuged at the same speed for 1 minute. Then, the column was transferred to a clean microcentrifuge tube, and 50 µL of DNA Elution Buffer was added directly onto the column matrix. Following a 5-minute incubation at room temperature, the tube was centrifuged at 8,000 rpm for 1 minute to elute the DNA. The spin column was discarded, and the eluted DNA was stored at −20°C.

DNA quantification and purity assessment

The DNA concentration was measured spectrophotometrically at 260 nm. Briefly, 495 µL of 10 mM Tris-Cl (pH 7.0) buffer was transferred to two cuvettes (500 µL capacity) and used to blank the spectrophotometer. To the second cuvette, 5 µL of DNA sample was added, thoroughly mixed with the buffer, and its absorbance at 260 nm (A_{260}) was recorded and used to calculate its concentration as follows:

$$\text{DNA concentration } (\mu\text{g/ml}) = \text{spectrophotometric conversion} \times (A_{260} - \text{correction factor}) \times \text{dilution factor}$$

Where spectrophotometric conversion = 50 µg/mL for dsDNA, correction factor = 0.012, and dilution factor = 100.

Samples with concentrations ≥50 ng/µL were used for further analysis.

Moreover, the purity of DNA was assessed by measuring absorbance at 280 nm (A280). The ratio $\frac{A_{260}}{A_{280}}$ was calculated to determine purity, with values ≥1.80 considered acceptable for PCR applications.

Polymerase Chain Reaction (PCR)

PCR amplification was performed using the SimpliAmp™ Thermal Cycler (Applied Biosystems, Thermo Fisher Scientific, Germany). The reaction volume was set to 25 µL and consisted of 10 µM Forward Primer (0.5 µL), 10 M Reverse Primer (0.5 µL), template DNA (2.0 µL), Taq 2X Master Mix (12.5 µL), and Nuclease-free water (9.5 µL). Primers targeting specific resistance genes (*vanA*, *vanB*, and *VanC*) were used to amplify products of defined sizes, as shown in Table 1. Annealing temperatures for each primer pair were optimised (54°C for *vanA*, *vanB,* and *vanC*). The PCR conditions were as summarised in Table 2:

Table 1: Target genes and their respective primers

Target gene	Primer Sequence (5' to 3')		Product size
van A	F	CATGAATAGAATAAAAGTTGCAATA	1000bp-1050bp
	R	CCCCTTTAACGCTAATACGATCAA	
van B	F	GTGACAAACCGGAGGCGAGGA	350 - 400bp
	R	CCGCCATCCTCCTGCAAAAAA	
van C	F	GAAAGACAACAGGAAGACCGC	700 - 750bp
	R	ATCGCATCACAAGCACCAATC	

Table 2: PCR conditions

Step	Temperature	Time	Cycles
Initial Denaturation	94°C	5 minutes	1
Denaturation	94°C	30 seconds	35
Annealing	54°C	30 seconds	35
Extension	72°C	30 seconds	35
Final Extension	72°C	5 minutes	1
Hold	4°C	Infinite	-

Gel electrophoresis

DNA amplicons were resolved using 1% (w/v) agarose gel electrophoresis. To prepare the gel, 0.5 g of agarose was dissolved in 50 mL of 1X TAE buffer by heating in a microwave. After

cooling to approximately 50°C, 2 µL of ethidium bromide was added for nucleic acid staining. The gel was poured into a tray fitted with an 8-well comb and allowed to set for 30 minutes. The DNA samples were prepared by mixing 10 µL of each PCR product with 2 µL of loading dye. A 5 µL DNA ladder (New England Biolabs) was loaded into the first well as a size marker. The gel was run at 120 V for 30 minutes using a Cleaver Scientific electrophoresis system (UK). Images were captured and documented using the gelLITE Gel Documentation System (Cleaver Scientific, UK).

Data analysis

Data were analysed using GraphPad Prism statistical software (version 10.3). Descriptive statistics, including frequencies and percentages, were used to summarise the patient demographics and diversity of bacterial isolates. Antibiotic resistance patterns were presented in tabular format to highlight trends and critical findings.

Ethical considerations

Ethical approval was obtained from the KTRH Ethics and Research Committee, University of Eastern Africa Baraton (REC: UEAB/ISERC/08/05/2024). Written informed consent was obtained from all participants or their legal guardians. Confidentiality of patient information was maintained throughout the study, and all procedures adhered to the Declaration of Helsinki principles.

Results

Socio-demographic and clinical characteristics of study patients

In this study, most patients from which bacterial isolates were obtained were male (53.02 %) (Table 3). It was observed that most of the patients were aged between 31 and 40 years (22.15 %), and avulsions constituted most of the wound types accounting for 34 %, while burns were the least frequent wounds (5 %) in the patients (Table 3). Moreover, the lower limb wounds were the most common (42.29 %) compared to upper limb wounds which accounted for about one third (33.56 %) (Table 3).

Table 3: Socio-demographic and clinical characteristics of study participants

Characteristics	Categories	Frequency (n)	%
Sex	Female	70	46.98
	Male	79	53.02
	0-10	9	6.04

Characteristics	Categories	Frequency (n)	%
	11-20	19	12.75
	21-30	22	14.77
	31-40	33	22.15
	41-50	18	12.08
Age (Years)	51-60	21	14.09
	61-70	11	7.38
	71-80	16	10.74
Wound type	Avulsions	50	34.00
	Abrasion	37	25.00
	Laceration	32	21.00
	Burns	7	5.00
	Punctures	23	15.00
	Trunk	36	24.16
Anatomic site	Upper Limb	50	33.56
	Lower Limb	63	42.29
**Total**		**149**	**100**

Bacterial strains isolated from wound swab cultures

This study noted that most of the bacterial isolates from the wound swabs included *E. coli* (27.97 %), *E. gergoviae* (16.95 %), and *S. aureus* and *S. xylosus* (15.25 %) (Table 4). Conversely, the least frequent isolates included *S. intermedius, E. aerogenes, P. vulgaris, P. mirabillis*, and *P. haemolytica*, standing for a paltry 0.85 % (Table 4).

Table 4: Bacterial strains isolated from wound swab cultures

Bacterial Isolate	Frequency (n)	Percentage (%)
Enterobacter gergoviae	20	16.95
Staphylococcus xylosus	18	15.25
Staphylococcus aureus	18	15.25
Staphylococcus intermedius	1	0.85
Escherichia coli	33	27.97
Serratia liquefaciens	5	4.24
Serratia odorifera 1	4	3.39
Enterobacter sakazaldi	2	1.69
Enterobacter aerogenes	1	0.85
Enterobacter cloacae	2	1.69
Proteus vulgaris	1	0.85
Proteus mirabillis	1	0.85
klebsiella ornithinofytica	2	1.69
Pausteurella haemolytica	1	0.85
Aeromonas hydrophila 1	2	1.69
Yersinia enterocolihca	2	1.69
Burkholderia pseudomallei	3	2.54
Pseudomonas aeruginosa	2	1.69
**Total number of isolates**	**118**	**100.0%**

Resistance of the bacterial isolates to various antibiotics

Resistance to ACCESS antibiotics

This study assessed the antibiotic resistance of 118 bacterial isolates against the ACCESS category of antibiotics including ampicillin, tetracycline, nitrofurantoin, nalidixic acid, streptomycin, cotrimoxazole-trimethoprim, and gentamycin, commonly used to treat wound infections [34], following CLSI guidelines [31]. Overall, the results revealed a considerably higher resistance of the isolated bacteria to Cotrimoxazole-Trimethoprim (97%) and Ampicillin (92%), with significant resistance observed to Nitrofurantoin (72%), Streptomycin (85%), and Nalidixic Acid (68%) (Table 5). Lower resistance rates were noted for Tetracycline (56%) and Gentamicin (49%) (Table 5).

Notably, as the results in Table 5 indicate, *E. gergoviae* exhibited widespread resistance, including 100% to Streptomycin and Cotrimoxazole-Trimethoprim, and 95% to Ampicillin. Similarly, *E. coli* (n=33) displayed high resistance to Cotrimoxazole-Trimethoprim (97%) and Ampicillin (93.9%), with moderate resistance to Gentamicin (57.6%) (Table 5). Besides, *S. aureus* (n=18) showed notable resistance to Cotrimoxazole-Trimethoprim (88.9%) and Streptomycin (66.7%), while *S. xylosus* (n=18) exhibited 100% resistance to Cotrimoxazole-Trimethoprim and 77.8% to Nalidixic Acid as shown in Table 5.

Other less frequent isolates such as *P. vulgaris, P. mirabilis, Y. enterocolitica*, and *B. pseudomallei* uniformly resisted multiple antibiotics (100%), highlighting multidrug resistance. Rare strains like *E. sakazakii, A. hydrophila*, and *P. aeruginosa* also showed higher resistance to multiple ACCESS antibiotics as shown in Table 5.

Table 5: Frequency of resistance to ACESS Antibiotics by the bacterial isolates

Bacterial Isolate	n	Proportion of resistant bacterial isolates to the ACCESS category of antibiotics						
		**AMP**	**TETRA**	**NITRO**	**NDA**	**STREP**	**COT**	**GENTA**
Enterobacter gergoviae	20	19 (95.0%)	14 (70.0%)	16 (80.0%)	12 (60.0%)	20 (100.0%)	20 (100.0%)	12 (60.0%)
Staphylococcus xylosus	18	16 (88.9%)	8 (44.4%)	13 (72.2%)	14 (77.8%)	13 (72.2%)	18 (100.0%)	7 (38.9%)
Staphylococcus aureus	18	14 (77.8%)	7 (38.9%)	9 (50.0%)	11 (61.1%)	12 (66.7%)	16 (88.9%)	6 (33.3%)
Staphylococcus intermedius	1	1 (100.0%)	0 (0.0%)	0 (0.0%)	1 (100.0%)	0 (0.0%)	1 (100.0%)	0 (0.0%)
Escherichia coli	33	31(93.9%)	22 (66.7%)	23 (69.7%)	23 (69.7%)	29 (87.9%)	32 (97.0%)	19 (57.6%)
Serratia liquefaciens	5	5 (100.0%)	2 (40.0%)	3 (60.0%)	4 (80.0%)	4 (80.0%)	5 (100.0%)	2 (40.0%)
Serratia odorifera 1	4	4 (100.0%)	2 (50.0%)	2 (50.0%)	2 (50.0%)	4 (100.0%)	4 (100.0%)	1 (25.0%)
Enterobacter sakazaldi	2	2 (100.0%)	2 (100.0%)	2 (100.0%)	1 (50.0%)	2 (100.0%)	1 (50.0%)	1 (50.0%)
Enterobacter aerogenes	1	1 (100.0%)	1 (100.0%)	1 (100.0%)	0 (0.0%)	1(100.0%)	1 (100.0%)	0 (0.0%)
Enterobacter cloacae	2	2 (100.0%)	0 (0.0%)	2 (100.0%)	1 (50.0%)	1(50.0%)	2 (100.0%)	1 (50.0%)
Proteus vulgaris	1	1 (100.0%)	1 (100.0%)	1 (100.0%)	1 (100.0%)	1(100.0%)	1 (100.0%)	1 (100.0%)
Proteus mirabillis	1	1 (100.0%)	1 (100.0%)	1 (100.0%)	1 (100.0%)	1(100.0%)	1 (100.0%)	1 (100.0%)
klebsiella ornithinofytica	2	2 (100.0%)	1 (50.0%)	2 (100.0%)	2 (100.0%)	2(100.0%)	2 (100.0%)	1 (50.0%)
Pausteurella haemolytica	1	1 (100.0%)	0 (0.0%)	1 (100.0%)	1 (100.0%)	1 (100.0%)	1 (100.0%)	1 (100.0%)
Aeromonas hydrophila 1	2	2 (100.0%)	0 (0.0%)	2 (100.0%)	1 (50.0%)	2 (100.0%)	2 (100.0%)	0 (0.0%)
Yersinia enterocolihca	2	2 (100.0%)	2 (100.0%)	2 (100.0%)	2 (100.0%)	2 (100.0%)	2 (100.0%)	2 (100.0%)
Burkholderia pseudomallei	3	3 (100.0%)	2 (67.0%)	3 (100.0%)	2 (67.0%)	3 (100.0%)	3 (100.0%)	2 (67.0%)
Pseudomonas aeruginosa	2	2 (100.0%)	1 (50.0%)	2 (100.0%)	1 (50.0%)	2 (100.0%)	2 (100.0%)	1 (50.0%)
Total	118	109(92.0%)	66 (56.0%)	85 (72.0%)	80 (68.0%)	100(85.0%)	114(97.0%)	58 (49.0%)

The values in parenthesis represent the percentage of bacterial isolates which showed resistance to respective antibiotics based on the CLSI guidelines [35]. AMP: Ampicillin; TETRA: Tetracycline; NITRO: Nitrofurantoin; NDA: Nalidixic acid; STREP: Streptomycin; COT: Cotrimoxazole-Trimethoprim; GENTA: Gentamycin.

Resistance to Watch and Reserve antibiotics

This study also evaluated resistance patterns in 118 bacterial isolates against Watch and Reserve antibiotics, including Ofloxacin, Ceftriaxone, Vancomycin, and Linezolid [34]. Resistance to these critical antibiotics was observed across both Gram-positive and Gram-negative isolates, underscoring the growing threat of antimicrobial resistance. Among Gram-positive isolates (n=37), resistance to Ofloxacin, Ceftriaxone, Vancomycin, and Linezolid was 18%, 32%, 29%, and 24%, respectively (Table 6). Notably, *S. aureus* (n=18) exhibited moderate resistance, particularly to Vancomycin (33%) and Ceftriaxone (27%), while *S. xylosus* (n=18) demonstrated similar resistance rates to Ceftriaxone (33%) and Vancomycin (26%) (Table 6). However, *S. intermedius* (n=1) showed complete resistance to Ceftriaxone (100%), though it was susceptible to other antibiotics (Table 6).

For the Gram-negative isolates (n=81), the overall resistance rates to Ofloxacin and Ceftriaxone were 29% and 33%, respectively (Table 6). In this study, *E. coli* (n=33) exhibited moderate resistance to Ofloxacin (18%) and Ceftriaxone (24%) (Table 6). Higher resistance rates to Ofloxacin and Ceftriaxone were observed in *E. gergoviae* (40% and 35%), *S. liquefaciens* (40% and 60%), and *B. pseudomallei* (66% and 33%), respectively as shown in Table 6. Alarming levels of resistance to Ofloxacin and Ceftriaxone were noted in *P. aeruginosa* (50% and 100%) and *E. sakazakii* (50% and 100%) (Table 6). While *E. cloacae* displayed complete resistance to Ofloxacin (100%), other isolates, such as *P. vulgaris* and *P. haemolytica*, remained susceptible (Table 6).

Table 6: Proportion of bacterial isolates resistant to WATCH and RESEARVE antibiotics

**Strain type of isolate**	**n**	**Proportion of resistance isolates to watch and reserve antibiotics**			
**Gram-positive**		**Ofloxacin**	**Ceftriaxone**	**Vancomycin**	**Linezolid**
Staphylococcus aureus	18	3(16)	5(27)	6(33)	5(27)
Staphylococcus xylosus	18	4(22)	6(33)	5(26)	4(21)
Staphylococcus intermedius	1	0(0)	1(100)	0(0)	0(0)
**Total**	**37**	**7(18)**	**12(32)**	**11(29)**	**9(24)**
**Gram-positive**					
Escherichia coli	33	6(18)	8(24)	NA	NA
Enterobacter gergoviae	20	8(40)	7(35)	NA	NA

Sarratia liquefaciens	5	2(40)	3(60)	NA	NA
Serratia odorifera	4	1(25)	1(25)	NA	NA
Burkholderia pseudomallei	3	2(66)	1(33)	NA	NA
Pseudomonas aeruginosa	2	1(50)	2(100	NA	NA
Aeromonas hydrophila 1	2	0(0)	0(0)	NA	NA
Klebsiella ornithinolytica	2	0(0)	0(0)	NA	NA
Yersinia enterocolitica	2	1(50)	1(50)	NA	NA
Enterobacter sakazakii	2	1(50)	2(100)	NA	NA
Enterobacter cloacae	2	2(100)	1(50)	NA	NA
Enterobacter aerogenes	1	0(0)	0(0)	NA	NA
Proteus vulgaris	1	0(0)	0(0)	NA	NA
Proteus mirabillis	1	0(0)	1(100)	NA	NA
Pausteurella haemolytica	1	0(0)	0(0)	NA	NA
**Total**	**81**	**24(29)**	**27(33)**	NA	NA

NA: Not assessed in this study

Vancomycin resistant genes

Considering that Staphylococcus species are frequently linked to wound infections, the presence of three genes associated with Vancomycin resistance were determined in this study. The results showed the presence of *Van*A in one *S. aureus* isolate (Figure 1a), VanB in one isolate of *S. aureus* and *S. xylosus* (Figure 1b), and VanC in four *S. aureus* and two *S. xylosus* isolates (Figure 1c), respectively.

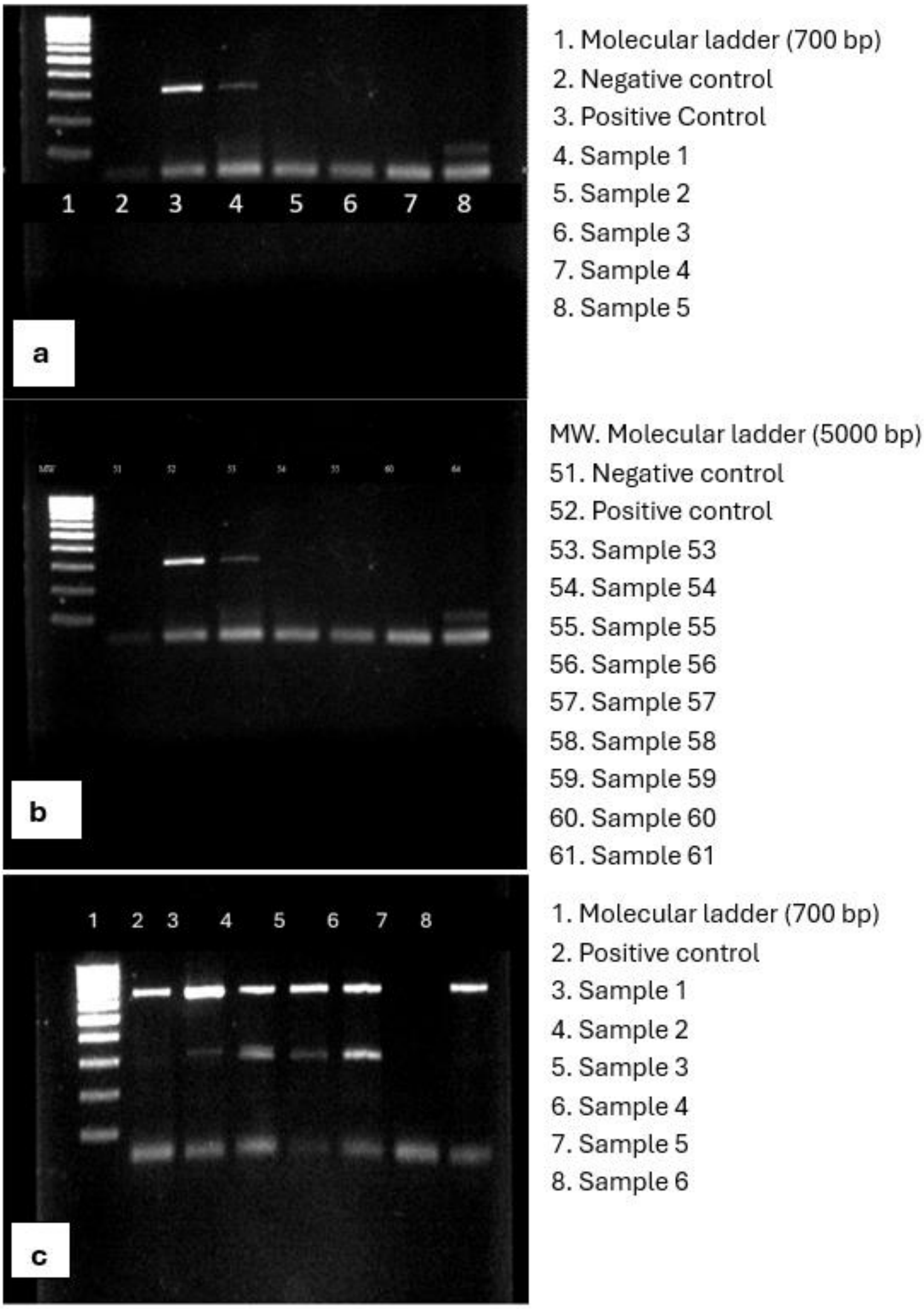

Figure 1: Electropherogram showing the detection of *Van*A gene in one *S. aureus* isolate (a), *Van*B in two isolates (one *S. aureus* and one *S. xylosus*) (b), and *Van*C in six isolates (four *S. aureus* and two *S. xylosus*) (C).

Discussion

This study investigated the prevalence of various bacterial pathogens from patient wound infections, their resistance to commonly used antibiotics, and the presence of resistance genes, particularly to critical Watch and Reserve antibiotics at Kisii Teaching and Referral Hospital. By providing a comprehensive understanding of the local AMR landscape, this study sought to inform targeted interventions, improve empirical treatment protocols, and contribute to the global efforts aiming at combating antimicrobial resistance (AMR) [18,36,37].

The predominance of male patients and those aged 31–40 years with various wound types requiring clinical management observed in this study suggests occupational or lifestyle factors that increase exposure to wound-causing injuries, highlighting the need for targeted prevention in high-risk groups [38]. The high frequency of lower limb wounds compared to upper limb wounds could be linked to anatomical vulnerability and environmental exposure, reinforcing the need for enhanced protective measures in occupational safety [39]. The high occurrence of avulsion wounds may indicate the need for early intervention protocols tailored to mechanically induced injuries [39,40]. Furthermore, the relatively low incidence of burns underscores the likely influence of regional environmental and occupational conditions, offering valuable context for prioritising wound management strategies [41]. These insights into patient demographics and wound characteristics are vital for developing region-specific healthcare policies and interventions, especially in resource-limited settings [42,43].

The observed diversity of bacterial isolates reflects the complex microbial landscape of wound infections in hospital settings [9,44]. The high prevalence of Gram-negative bacteria, especially *E. coli* and *E. gergoviae* suggests a significant role for faecal contamination or environmental sources in these infections, warranting stricter hygiene and sanitation protocols [45]. The significant presence of Gram-positive bacteria like *S. aureus* and *S. xylosus* emphasises their role in wound pathophysiology, potentially exacerbated by virulence factors such as toxin production and biofilm formation [46]. Besides, the low prevalence of rare isolates such as *P. mirabilis* and *P. haemolytica* highlights their opportunistic pathogenicity, often linked to immunocompromised hosts or specific environmental exposures [47]. These findings underline the importance of periodic surveillance of wound microbiota to capture emerging resistance patterns and inform empirical treatment choices. Additionally, recognising the unique microbial profiles in specific wound types may facilitate the development of targeted therapeutic and prophylactic interventions, ultimately improving patient outcomes.

The alarming resistance rates to ACCESS antibiotics observed in this study signify the limitations of first-line therapies and the escalating threat of antimicrobial resistance [48]. The near-universal resistance of *E. gergoviae* and *E. coli* to Cotrimoxazole-Trimethoprim and Ampicillin underscores the urgency of revising empirical treatment protocols [49,50]. Resistance to Nitrofurantoin, Streptomycin, and Nalidixic Acid among other antibiotics further compounds the therapeutic challenge, especially for community-acquired infections [51]. Such widespread resistance necessitates urgent investment in antimicrobial stewardship programmes to preserve the efficacy of existing antibiotics [52]. The moderate resistance to Tetracycline and Gentamicin, though relatively lower, underscores their potential utility in some cases while highlighting the need for continuous monitoring to prevent escalation [53]. Integrating local resistance data into clinical guidelines will optimise treatment regimens and minimise therapeutic failures in regions with similar microbial profiles [54]. Furthermore, the observed resistance patterns call for the adoption of precision medicine approaches that include pathogen-specific susceptibility testing to guide effective treatment [55].

The emergence of resistance to Watch and Reserve antibiotics, including Ofloxacin and Ceftriaxone, among both Gram-positive and Gram-negative isolates, is particularly concerning [48]. These antibiotics, often regarded as last-resort treatments, are critical for managing multidrug-resistant infections [56]. Resistance to Vancomycin and Linezolid among Gram-positive isolates, including *S. aureus*, could lead to increased morbidity and mortality, especially in resource-constrained settings where alternative treatments may be limited [55,57]. The high resistance observed in *P. aeruginosa* and *E. sakazakii* to Ofloxacin and Ceftriaxone underscores the need for strict antibiotic use policies to limit the emergence of resistance in nosocomial settings [56,58]. These findings further highlight the importance of infection prevention and control measures to reduce the spread of resistant strains [59]. The apparent cross-resistance among critical antibiotics raises the spectre of therapeutic dead-ends, necessitating policy-driven interventions that prioritise antibiotic conservation and effective stewardship programmes at all levels of care [60].

The molecular detection of Vancomycin resistance genes (*Van*A, *Van*B, *Van*C) in *S. aureus* and *S. xylosus* provides a crucial link between genotypic and phenotypic resistance [61]. The presence of these genes suggests horizontal gene transfer as a potential driver of resistance, raising concerns about the genetic adaptability of bacterial pathogens [62,63]. These findings underscore the need for molecular surveillance in AMR monitoring programmes to track resistance evolution [55]. Identifying resistance genes also offers opportunities for the

development of rapid diagnostic tools to detect resistant pathogens and implement timely interventions [57]. Additionally, understanding the genetic mechanisms underpinning resistance could inform the discovery of novel therapeutic targets, thereby bolstering efforts to counteract the AMR crisis.

From a research perspective, these findings underscore the importance of prioritising the development of novel antimicrobial agents and exploring alternative therapeutic approaches. The high prevalence of multidrug-resistant pathogens calls for innovative strategies such as combination therapies, bacteriophage applications, and immunotherapies [56]. Future studies should also investigate the role of local environmental and healthcare practices in driving AMR, as such insights are critical for designing tailored interventions. Understanding these contextual factors will help in designing interventions that are both effective and sustainable in diverse clinical settings. The integration of advanced omics technologies into routine diagnostics may further refine our understanding of resistance mechanisms and pathogen-host interactions, thereby advancing personalised medicine in infectious disease management.

This study highlights the need to strengthen antimicrobial stewardship and infection control policies, especially in tertiary healthcare institutions like Kisii Teaching and Referral Hospital. Implementing national surveillance systems to capture resistance patterns will support evidence-based decision-making and resource allocation [64]. Public awareness campaigns on the judicious use of antibiotics can complement these measures, reducing the demand for inappropriate prescriptions [54]. Additionally, fostering collaborations between healthcare facilities, researchers, and policymakers can help address the multifaceted challenges posed by AMR. Strengthening laboratory capacity to routinely conduct susceptibility testing and monitor resistance trends is essential for effective policy implementation and sustained AMR mitigation.

Limitations

This study has several limitations that warrant consideration. Firstly, the sample size, though sufficient for initial insights, may not fully capture the microbial diversity and resistance patterns in other healthcare settings or broader populations. Secondly, the study was conducted in a single tertiary hospital, potentially limiting the generalisability of findings to other regions with differing healthcare practices and environmental factors. Thirdly, the reliance on phenotypic methods for antimicrobial susceptibility testing, while robust, may not detect all resistance mechanisms, underscoring the need for complementary molecular analyses. Lastly,

the cross-sectional design precludes the assessment of temporal trends in resistance patterns, which could provide a more dynamic understanding of antimicrobial resistance. Future research should address these limitations through multicentre studies, larger sample sizes, and longitudinal designs.

Conclusions and recommendations

This study showed the presence of a wide variety of bacterial strain strains in infected wounds of patients at Kisii Teaching and Referral Hospital. Besides the findings underscore the significant burden of multidrug-resistant pathogens in wound infections, demonstrated by high resistance rates to both ACCESS antibiotics, such as Cotrimoxazole-Trimethoprim (97%) and Ampicillin (92%), and critical Watch and Reserve antibiotics, including Vancomycin (29%) and Ceftriaxone (33%). The detection of resistance genes like *VanA*, *VanB*, and *VanC* in *Staphylococcus* species highlights the genetic mechanisms underpinning resistance and the potential for horizontal gene transfer. Socio-demographic and clinical factors, such as the predominance of male patients, lower limb wounds, and avulsions, may further inform targeted prevention strategies. These findings emphasise the urgent need for evidence-based antimicrobial stewardship to mitigate therapeutic failures and protect patient outcomes in high-burden settings.

Strengthening infection prevention and control measures, such as standardised wound care protocols and enhanced hygiene practices, is critical to curbing the spread of resistant pathogens. Policymakers should prioritise the establishment of robust AMR surveillance systems and the integration of local resistance data into clinical guidelines to optimise empirical therapy. Public education campaigns to promote the rational use of antibiotics can complement these measures. Future research should focus on developing alternative therapeutic strategies, such as combination therapies or bacteriophage applications, and investigating the environmental and healthcare-related drivers of AMR to inform sustainable interventions. These efforts can significantly contribute to combating AMR and improving patient outcomes in resource-constrained healthcare settings.

Declarations

Availability of data and materials

All the data and materials used in this study are included in this manuscript; however, the authors can provide additional information upon reasonable request.

Competing Interests

There are no perceived, financial, or otherwise conflicts of interest with respect to this publication.

Funding

This study did not receive any formal funding from any research funding or granting entity in public, private, or not for profit organisations.

Authors' contributions

Samson Onsando formulated the research idea and designed the experiments under the guidance of Andrew Nyerere and Moses Njire. Samson Onsando performed the experiments, analysed the data, and wrote the manuscript with the supervision of Andrew Nyerere, Moses Njire, Wycliffe Masanta, and Gervason Moriasi. All authors reviewed and approved the final draft for submission and publication.

Acknowledgements

We are grateful for the technical assistance provided by Mr. Kiyondi, Mr. D. Ounga and Mrs. R. Anyango of Kisii Teaching, and Referral Hospital (Microbiology laboratory).

References

1. Falcone M, De Angelis B, Pea F, Scalise A, Stefani S, Tasinato R, et al. Challenges in the management of chronic wound infections. J Glob Antimicrob Resist. 2021;26: 140–147. doi:10.1016/j.jgar.2021.05.010

2. Rojas-Gutierrez E, Vilar-Compte D. An Overview of Surgical Site Infection in Low- and Middle-Income Countries: the Role of Recent Guidelines, Limitations, and Possible Solutions. Curr Treat Options Infect Dis. 2019;11: 300–316. doi:10.1007/s40506-019-00198-1

3. Ologunde R, Maruthappu M, Shanmugarajah K, Shalhoub J. Surgical care in low and middle-income countries: Burden and barriers. International Journal of Surgery. 2014;12: 858–863. doi:10.1016/j.ijsu.2014.07.009

4. Kirkup BC. Bacterial Strain Diversity Within Wounds. Adv Wound Care (New Rochelle). 2015;4: 12–23. doi:10.1089/wound.2014.0560

5. Uberoi A, McCready-Vangi A, Grice EA. The wound microbiota: microbial mechanisms of impaired wound healing and infection. Nat Rev Microbiol. 2024;22: 507–521. doi:10.1038/s41579-024-01035-z

6. Medical Education Partnership Ltd. Wound Infection in Clinical Practice: An International Consensus. Int Wound J. 2008;5. doi:10.1111/j.1742-481X.2008.00488.x

7. Darwin E, Tomic-Canic M. Healing Chronic Wounds: Current Challenges and Potential Solutions. Curr Dermatol Rep. 2018;7: 296–302. doi:10.1007/s13671-018-0239-4

8. de Mestral C, Nathens AB. Prevention, Diagnosis, and Management of Surgical Site Infections. Crit Care Clin. 2013;29: 887–894. doi:10.1016/j.ccc.2013.06.009

9. Ding X, Tang Q, Xu Z, Xu Y, Zhang H, Zheng D, et al. Challenges and innovations in treating chronic and acute wound infections: from basic science to clinical practice. Burns Trauma. 2022;10. doi:10.1093/burnst/tkac014

10. Dobson GP. Trauma of major surgery: A global problem that is not going away. International Journal of Surgery. 2020;81: 47–54. doi:10.1016/j.ijsu.2020.07.017

11. De Waele J, De Bus L. How to treat infections in a surgical intensive care unit. BMC Infect Dis. 2014;14: 193. doi:10.1186/1471-2334-14-193

12. Qadan M, Cheadle WG. Common Microbial Pathogens in Surgical Practice. Surgical Clinics of North America. 2009;89: 295–310. doi:10.1016/j.suc.2008.09.002

13. Puca V, Marulli RZ, Grande R, Vitale I, Niro A, Molinaro G, et al. Microbial Species Isolated from Infected Wounds and Antimicrobial Resistance Analysis: Data Emerging from a Three-Years Retrospective Study. Antibiotics. 2021;10: 1162. doi:10.3390/antibiotics10101162

14. Peters A, Schmid MN, Parneix P, Lebowitz D, de Kraker M, Sauser J, et al. Impact of environmental hygiene interventions on healthcare-associated infections and patient colonization: a systematic review. Antimicrob Resist Infect Control. 2022;11: 38. doi:10.1186/s13756-022-01075-1

15. Chirca I. The Hospital Environment and its Microbial Burden: Challenges and Solutions. Future Microbiol. 2019;14: 1007–1010. doi:10.2217/fmb-2019-0140

16. Ahmed SK, Hussein S, Qurbani K, Ibrahim RH, Fareeq A, Mahmood KA, et al. Antimicrobial resistance: Impacts, challenges, and future prospects. Journal of Medicine, Surgery, and Public Health. 2024;2: 100081. doi:10.1016/j.glmedi.2024.100081

17. Naghavi M, Vollset SE, Ikuta KS, Swetschinski LR, Gray AP, Wool EE, et al. Global burden of bacterial antimicrobial resistance 1990–2021: a systematic analysis with forecasts to 2050. The Lancet. 2024;404: 1199–1226. doi:10.1016/S0140-6736(24)01867-1

18. Mittal AK, Bhardwaj R, Mishra P, Rajput SK. Antimicrobials Misuse/Overuse: Adverse Effect, Mechanism, Challenges and Strategies to Combat Resistance. Open Biotechnol J. 2020;14: 107–112. doi:10.2174/1874070702014010107

19. Moyo P, Moyo E, Mangoya D, Mhango M, Mashe T, Imran M, et al. Prevention of antimicrobial resistance in sub-Saharan Africa: What has worked? What still needs to be done? J Infect Public Health. 2023;16: 632–639. doi:10.1016/j.jiph.2023.02.020

20. Ita T, Luvsansharav U-O, Smith RM, Mugoh R, Ayodo C, Oduor B, et al. Prevalence of colonization with multidrug-resistant bacteria in communities and hospitals in Kenya. Sci Rep. 2022;12: 22290. doi:10.1038/s41598-022-26842-3

21. Kimani R, Wakaba P, Kamita M, Mbogo D, Mutai W, Ayodo C, et al. Detection of multidrug-resistant organisms of concern including Stenotrophomonas maltophilia and Burkholderia cepacia at a referral hospital in Kenya. PLoS One. 2024;19: e0298873. doi:10.1371/journal.pone.0298873

22. Maveke SM, Aboge GO, Kanja LW, Mainga AO, Gachau N, Muchira BW, et al. Phenotypic and Genotypic Characterization of Extended Spectrum Beta-Lactamase-Producing Clinical Isolates of Escherichia coli and Klebsiella pneumoniae in Two Kenyan Facilities: A National Referral and a Level Five Hospital. Int J Microbiol. 2024;2024. doi:10.1155/2024/7463899

23. Dohoo L, Martin W, Stryhn H. Veterinary Epidemiologic Research. S Margaret McPike, editor. Prince Edward Island, Canada, CIA 4P3: National Library of Canada Cataloguing in Publication; 2003.

24. Sonda T, Kumburu H, van Zwetselaar M, Alifrangis M, Mmbaga BT, Lund O, et al. Molecular epidemiology of virulence and antimicrobial resistance determinants in Klebsiella pneumoniae from hospitalised patients in Kilimanjaro, Tanzania. European Journal of Clinical Microbiology and Infectious Diseases. 2018;37: 1901–1914. doi:10.1007/s10096-018-3324-5

25. Fernandez R, Green HL, Griffiths R, Atkinson RA, Ellwood LJ. Water for wound cleansing. Cochrane Database of Systematic Reviews. 2022;2022. doi:10.1002/14651858.CD003861.pub4

26. Li S, Renick P, Senkowsky J, Nair A, Tang L. Diagnostics for Wound Infections. Adv Wound Care (New Rochelle). 2021;10: 317–327. doi:10.1089/wound.2019.1103

27. Clinical Laboratory Standards Institute (CLSI). Clinical Laboratory Standards Institute (CLSI) - CLSI M100 ED30:2020. 2020. Available: http://em100.edaptivedocs.net/GetDoc.aspx?doc=CLSI%20M100%20ED30:2020&sbssok=CLSI%20M100%20ED30:2020%20SECTION%20COMMITTEE%20MEMBERSHIP%20[NEXT]

28. Bonnet M, Lagier JC, Raoult D, Khelaifia S. Bacterial culture through selective and non-selective conditions: the evolution of culture media in clinical microbiology. New Microbes New Infect. 2020;34: 100622. doi:10.1016/j.nmni.2019.100622

29. Cheesbrough M. District Laboratory Practice in Tropical Countries. 2nd ed. Cheesbrough M, editor. Cambrige: Cambridge University Press; 2010. doi:10.1017/CBO9780511581304

30. Nakasone I, Kinjo T, Yamane N, Kisanuki K, Shiohira CM. Laboratory-based evaluation of the colorimetric VITEK-2 Compact system for species identification and of the

Advanced Expert System for detection of antimicrobial resistances: VITEK-2 Compact system identification and antimicrobial susceptibility testing. Diagn Microbiol Infect Dis. 2007;58: 191–198. doi:10.1016/j.diagmicrobio.2006.12.008

31. Lewis JS., Weinstein MP., Bobenchik AM., Cameau Shelley, Cullen SK., Dingle Tanis, et al. M100 : performance standards for antimicrobial susceptibility testing. Clinical and Laboratory Standards Institute; 2023.

32. Clinical Laboratory Standards Institute (CLSI). Analysis and Presentation of Cumulative Antimicrobial Susceptibility Test Data; Approved CLSI Guideline document M39-A4. Fifth Edition. Janet A. Hindler MMF, Patricia J. Simner PD, April Abbott P (ABMM), Faiza H. Benahmed M, Tanaya Bhowmick M, Sanchita Das MD, et al., editors. PA: Wayne PA; 2022.

33. Zymo Research. Quick-DNA ™ Miniprep Kit: Rapid and simple isolation of ultra-pure DNA from biological liquids and cells. 2024.

34. WHO. The WHO The WHO A AWaRe (Access Access, Watch Watch, Reserve Reserve) antibiotic book antibiotic book. 2022nd ed. Geneva: World Health Organisation; 2022.

35. Lewis JS., Weinstein MP., Bobenchik AM., Cameau Shelley, Cullen SK., Dingle Tanis, et al. M100 : performance standards for antimicrobial susceptibility testing. 33rd ed. Clinical and Laboratory Standards Institute; 2023.

36. Calvo-Villamañán A, San Millán Á, Carrilero L. Tackling AMR from a multidisciplinary perspective: a primer from education and psychology. International Microbiology. 2022;26: 1. doi:10.1007/S10123-022-00278-1

37. Mudenda S, Chabalenge B, Daka V, Mfune RL, Salachi KI, Mohamed S, et al. Global Strategies to Combat Antimicrobial Resistance: A One Health Perspective. Pharmacology & Pharmacy. 2023;14: 271–328. doi:10.4236/PP.2023.148020

38. Debelu D, Mengistu DA, Tolera ST, Aschalew A, Deriba W. Occupational-Related Injuries and Associated Risk Factors Among Healthcare Workers Working in Developing Countries: A Systematic Review. Health Serv Res Manag Epidemiol. 2023;10. doi:10.1177/23333928231192834

39. Hopkins A, Samuriwo R. Comparison of compression therapy use, lower limb wound prevalence and nursing activity in England: a multisite audit. J Wound Care. 2022;31: 1016–1028. doi:10.12968/jowc.2022.31.12.1016

40. Choi C, Lee SJ, Choo HJ, Lee IS, Kim SK. Avulsion injuries: an update on radiologic findings. Yeungnam Univ J Med. 2021;38: 289–307. doi:10.12701/yujm.2021.01102

41. Yakupu A, Zhang J, Dong W, Song F, Dong J, Lu S. The epidemiological characteristic and trends of burns globally. BMC Public Health. 2022;22: 1596. doi:10.1186/s12889-022-13887-2

42. Liosatos K, Tobiano G, Gillespie BM. Patient participation in surgical wound care in acute care settings: An integrative review. Int J Nurs Stud. 2024;157: 104839. doi:10.1016/j.ijnurstu.2024.104839

43. Gray TA, Wilson P, Dumville JC, Cullum NA. What factors influence community wound care in the UK? A focus group study using the Theoretical Domains Framework. BMJ Open. 2019;9: e024859. doi:10.1136/bmjopen-2018-024859

44. Bertesteanu S, Triaridis S, Stankovic M, Lazar V, Chifiriuc MC, Vlad M, et al. Polymicrobial wound infections: Pathophysiology and current therapeutic approaches. Int J Pharm. 2014;463: 119–126. doi:10.1016/j.ijpharm.2013.12.012

45. Aiesh BM, Natsheh M, Amar M, AbuTaha S, Qadi M, AbuTaha A, et al. Epidemiology and clinical characteristics of patients with healthcare-acquired multidrug-resistant Gram-negative bacilli: a retrospective study from a tertiary care hospital. Sci Rep. 2024;14: 3022. doi:10.1038/s41598-024-53596-x

46. Puca V, Marulli RZ, Grande R, Vitale I, Niro A, Molinaro G, et al. Microbial Species Isolated from Infected Wounds and Antimicrobial Resistance Analysis: Data Emerging from a Three-Years Retrospective Study. Antibiotics. 2021;10: 1162. doi:10.3390/antibiotics10101162

47. Qu X, Zhou J, Huang H, Wang W, Xiao Y, Tang B, et al. Genomic Investigation of Proteus mirabilis Isolates Recovered From Pig Farms in Zhejiang Province, China. Front Microbiol. 2022;13. doi:10.3389/fmicb.2022.952982

48. Okeke IN, de Kraker MEA, Van Boeckel TP, Kumar CK, Schmitt H, Gales AC, et al. The scope of the antimicrobial resistance challenge. The Lancet. 2024;403: 2426–2438. doi:10.1016/S0140-6736(24)00876-6

49. Bhosle A, Datey A, Chandrasekharan G, Singh D, Chakravortty D, Chandra N. A Strategic Target Rescues Trimethoprim Sensitivity in Escherichia coli. iScience. 2020;23: 100986. doi:10.1016/j.isci.2020.100986

50. Pouwels KB, Batra R, Patel A, Edgeworth JD, Robotham J V., Smieszek T. Will co-trimoxazole resistance rates ever go down? Resistance rates remain high despite decades of reduced co-trimoxazole consumption. J Glob Antimicrob Resist. 2017;11: 71–74. doi:10.1016/j.jgar.2017.07.013

51. Njenga J, Nyasinga J, Munshi Z, Muraya A, Omuse G, Ngugi C, et al. Genomic characterization of two community-acquired methicillin-resistant Staphylococcus aureus with novel sequence types in Kenya. Front Med (Lausanne). 2022;9. doi:10.3389/fmed.2022.966283

52. Cohn J, Mendelson M, Kanj SS, Shafiq N, Boszczowski I, Laxminarayan R. Accelerating antibiotic access and stewardship: a new model to safeguard public health. Lancet Infect Dis. 2024;24: e584–e590. doi:10.1016/S1473-3099(24)00070-7

53. Livorsi DJ, Drainoni ML, Reisinger HS, Nanda N, McGregor JC, Barlam TF, et al. Leveraging implementation science to advance antibiotic stewardship practice and research. Infect Control Hosp Epidemiol. 2022;43: 139–146. doi:10.1017/ICE.2021.480

54. Fwoloshi S, Chola U, Nakazwe R, Tatila T, Mateele T, Kabaso M, et al. Why local antibiotic resistance data matters – Informing empiric prescribing through local data collation, app design and engagement in Zambia. J Infect Public Health. 2023;16: 69–77. doi:10.1016/j.jiph.2023.11.007

55. Chinemerem Nwobodo D, Ugwu MC, Oliseloke Anie C, Al-Ouqaili MTS, Chinedu Ikem J, Victor Chigozie U, et al. Antibiotic resistance: The challenges and some emerging strategies for tackling a global menace. J Clin Lab Anal. 2022;36. doi:10.1002/jcla.24655

56. Jan B, Jan R, Afzal S, Ayoub M, Masoodi MH. Treatment Strategies to Combat Multidrug Resistance (MDR) in Bacteria. Non-traditional Approaches to Combat Antimicrobial Drug Resistance. Singapore: Springer Nature Singapore; 2023. pp. 79–100. doi:10.1007/978-981-19-9167-7_4

57. AbdAlhafiz AI, Elleboudy NS, Aboshanab KM, Aboulwafa MM, Hassouna NA. Phenotypic and genotypic characterization of linezolid resistance and the effect of antibiotic combinations on methicillin-resistant Staphylococcus aureus clinical isolates. Ann Clin Microbiol Antimicrob. 2023;22: 23. doi:10.1186/s12941-023-00574-2

58. Elias C, Moja L, Mertz D, Loeb M, Forte G, Magrini N. Guideline recommendations and antimicrobial resistance: the need for a change. BMJ Open. 2017;7: e016264. doi:10.1136/bmjopen-2017-016264

59. Knobloch MJ, McKinley L, Keating J, Safdar N. Integrating antibiotic stewardship and infection prevention and control programs using a team science approach. Am J Infect Control. 2021;49: 1072. doi:10.1016/J.AJIC.2021.01.020

60. Elias C, Moja L, Mertz D, Loeb M, Forte G, Magrini N. Guideline recommendations and antimicrobial resistance: the need for a change. BMJ Open. 2017;7: e016264. doi:10.1136/bmjopen-2017-016264

61. Li G, Walker MJ, De Oliveira DMP. Vancomycin Resistance in Enterococcus and Staphylococcus aureus. Microorganisms. 2022;11: 24. doi:10.3390/microorganisms11010024

62. Gardete S, Tomasz A. Mechanisms of vancomycin resistance in Staphylococcus aureus. Journal of Clinical Investigation. 2014;124: 2836–2840. doi:10.1172/JCI68834

63. Stogios PJ, Savchenko A. Molecular mechanisms of vancomycin resistance. Protein Science. 2020;29: 654–669. doi:10.1002/pro.3819

64. WHO. Global Antimicrobial Resistance and Use Surveillance System (GLASS) Report. Geneva; 2021 Jun.
