# Supplementary material for: Diversity and antibiotic susceptibility profiles of bacterial isolates from wound infections in patients at the surgical unit of Kisii teaching and referral hospital, Kenya
Source: PLoS One. 2025 Jun 17;20(6):e0326048. doi: 10.1371/journal.pone.0326048 (PMC12173245; doi:10.1371/journal.pone.0326048)
Supplement: Attachment — Submitted filename: Response to Review Comments-Onsando et al-Plos One 12-4-25.pdf [file pone.0326048.s004.pdf]

PLOS ONE

Responses to Review Comments

Manuscript Title: **Diversity and antibiotic susceptibility profiles of bacterial isolates from wound infections in patients at the surgical unit of Kisii Teaching and Referral Hospital, Kenya**

Manuscript ID: PONE-D-25-04326

Dear Editors and Reviewers,

I heartly thank you for taking your valuable time to review our manuscript. Moreover, I appreciate your positive criticism, strong comments, and suggestions to improve the quality of our research article. We have carefully studied all the review comments and revised our manuscript accordingly. Evidently, the quality of our revised research article has improved significantly based on your critique and helpful suggestions. It is our hope that our revised manuscript now meets the standards and approval criteria for publication in this esteemed Journal of PLoS ONE, having addressed all the concerns. Please find our point-by-point responses to the reviewer's comments (highlighted yellow) and the author responses (indicated in green) below.

Reviewer 1

1. The authors have made tremendous improvements in the quality of the manuscript. I must commend all their efforts. However, there is still an issue with the sample size calculation which unfortunately still needs to be addressed. The formula is now correct however the prevalence (p) used in the formula should be the prevalence of Antimicrobial resistance among patients with wound infection rather than the prevalence of wound infection. Since you're exclusively studying patients who already have wound infections, Your sample size calculation should then focus on estimating antimicrobial resistance within that population of patients with wound infection. In this scenario, you should use the prevalence of antimicrobial resistance among patients with wound infections from prior studies, since resistance is a key outcome. Authors' response: Thank you so much for your keen review and helpful suggestion. We have now amended the prevalence to that of antimicrobial resistance among patients with wound infections and recalculated the sample size accordingly.
2. Similarly in the inclusion and exclusion criteria, there is no need to mention "consent" as a criteria for enrolment into a study. This is obvious. Its unethical to even enroll anyone in a research without consent'
   Authors' response: Thank you for this suggestion. Accordingly, we have expunged the last sentence in this section as it is unnecessary.
3. I noticed you have some minors as study subjects. It is important to include how you obtained "assent" from these participants and consent from their caregivers.
   Authors' response: Thank you for observation. For patients aged below 18 years, their parents or legal guardians provided informed consent according to ethical research guidelines.

****

Thank you so much for helping us to improve the quality our research article. We look forward to your feedback.
